# Association between small nucleolar RNA host gene expression and survival outcome of colorectal cancer patients: A meta-analysis based on PRISMA and bioinformatics analysis

**DOI:** 10.3389/fonc.2023.1094131

**Published:** 2023-02-20

**Authors:** Pei Luo, Jie Du, Yinan Li, Jilong Ma, Wenjun Shi

**Affiliations:** ^1^ Department of Gastroenterology, Qian Xi Nan Buyi and Miao Autonomous Prefecture People's Hospital, Xingyi, Guizhou, China; ^2^ Colorectal Surgery Department, Yunnan Cancer Hospital, The Third Affiliated Hospital of Kunming Medical University, Kunming, Yunnan, China

**Keywords:** lncRNA, SNHGs, colorectal cancer, prognosis, meta-analysis, bioinformatics analysis

## Abstract

**Introduction:**

Growing evidence shows that long non-coding RNA small nucleolar RNA host genes (lncRNA SNHGs) enact an pivotal regulatory roles in the shorter survival outcome of colorectal cancer (CRC). However, no research has systematically evaluated the correlation among lncRNA SNHGs expression and survival outcome of CRC. This research indented to screen whether exist potential prognostic effect of lncRNA SNHGs in CRC patientss using comprehensive review and meta-analysis.

**Methods:**

Systematic searches were performed from the six relevant databases from inception to October 20, 2022. The quality of published papers was evaluated in details. We pooled the hazard ratios (HR) with 95% confidence interval (CI) through direct or indirect collection of effect sizes, and odds ratios (OR) with 95% CI by collecting effect sizes within articles. Detailed downstream signaling pathways of lncRNA SNHGs were summarized in detail

**Results:**

25 eligible publications including 2,342 patients were finally included to appraise the association of lncRNA SNHGs with prognosis of CRC. Elevated lncRNA SNHGs expression was revealed in colorectal tumor tissues. High lncSNHG expression means bad survival prognosis in CRC patients (HR=1.635, 95% CI: 1.405–1.864, P<0.001). Additionally, high lncRNA SNHGs expression was inclined to later TNM stage (OR=1.635, 95% CI: 1.405–1.864, P<0.001), distant lymph node invasion, distant organ metastasis, larger tumor diameter and poor pathological grade. Begg's funnel plot test using the Stata 12.0 software suggested that no significant heterogeneity was found.

**Conclusion:**

Elevated lncRNA SNHGs expression was revealed to be positively correlated to discontented CRC clinical outcome and lncRNA SNHG may act as a potential clinical prognostic index for CRC patients.

## 1 Introduction

Colorectal cancer (CRC) seriously threatens human life and health, well-being and happiness quotient. According to 2021 cancer statistics, the annual incidence and mortality of CRC worldwide was ranked second and third in the world, respectively (1). With the development of medicine, various treatment options, for instance, radiotherapy, chemotherapy, biological targeted therapy and molecular biological therapy have been adopted in CRC therapy (2-4). Nevertheless, the five-year and ten-year survival rate of CRC patientss is unsatisfactory as before (5). While optimizing the strategies of combined radiotherapy and chemotherapy and targeted therapy and immunotherapy, researchers are also trying to hunt for splendid therapeutic targets to ameliorate the survival and prognosis of CRC cases.

Over the past ten years, mounting investigations have focalized long non-coding RNAs (lncRNA), a sort of small molecules of >200 nucleotide units without protein-coding functions, which enact a crucial part in the stride of CRC (6, 7). lncRNAs can affect the biological property of tumor cells at the cellular function level by affecting intracellular transduction pathways, such as cell proliferation, drug resistance, immune evasion and apoptosis (8, 9). Hence, lncRNAs could be used as underlying therapeutic targets and effective prognostic markers for tumor therapy.Small nucleolar RNA host genes (SNHGs), is a long non-coding RNA family, have been shown to be up-regulated in CRC, mounting publications suggests that lncRNA SNHG play increasingly prominent motivation in the biological properties of CRC cells (10, 11), for example, some researchers have uncovered that high SNHG expression could drive the growth, distant organ migration, invasion, and inhibition of apoptosis in CRC cells (12-15). Till date, there is no literature that evaluates the correlation between the lncRNA SNHG family and CRC prognosis. Results of some studies are inconsistent, and a single study has insufficient data. At the same time, we executed a comprehensively systematic assessment and meta-analysis to figure out the relation of the lncRNA SNHG family and CRC prognosis.

## 2 Materials and methods

### 2.1 Retrieval of studies

A systematical search of databases of PubMed (Medline), Embase, ISI Web of Knowledge, Springer, the Cochrane Library, Scopus, BioMed Central, ScienceDirect, Wanfang, Weipu, and China National Knowledge Internet was conducted from inception to November 1, 2022. Appropriate publications was searched in accordance with the detailed Mesh, including (“lnc SNHG” OR “long non-coding RNA SNHG” OR “Small nucleolar RNA host genes”) AND (“prognosis” OR “prognostic” OR “survival” OR “outcome”) based on PRISMA (The Preferred Reporting Items for Systematic Reviews and Meta-Analyses).

### 2.2 Inclusion and exclusion criteria

Studies were selected by two independent researchers. Inclusion criteria were as follows: (1) investigation of the association between SNHG expression and survival outcome, as well as clinical prognosis of CRC patients; (2) classification of patients into high and low expression groups in accordance with primary literature; (3) detection of SNHG expression level using validated techniques; (4) sufficient data to calculate odds ratio (OR) or hazard ratio (HR); and (5) studies written in English. Exclusion criteria were: (1) no investigation on the relationship between SNHG expression level and CRC prognosis different from a mere exploration of the involved molecular biological mechanisms; (2) reviews and meta-analyses, letters, animal studies, and conference proceedings; (3) insufficient data for prognostic analysis; and (4) duplicate studies.

### 2.3 Quality assessment of selected studies

**Figure 1:**
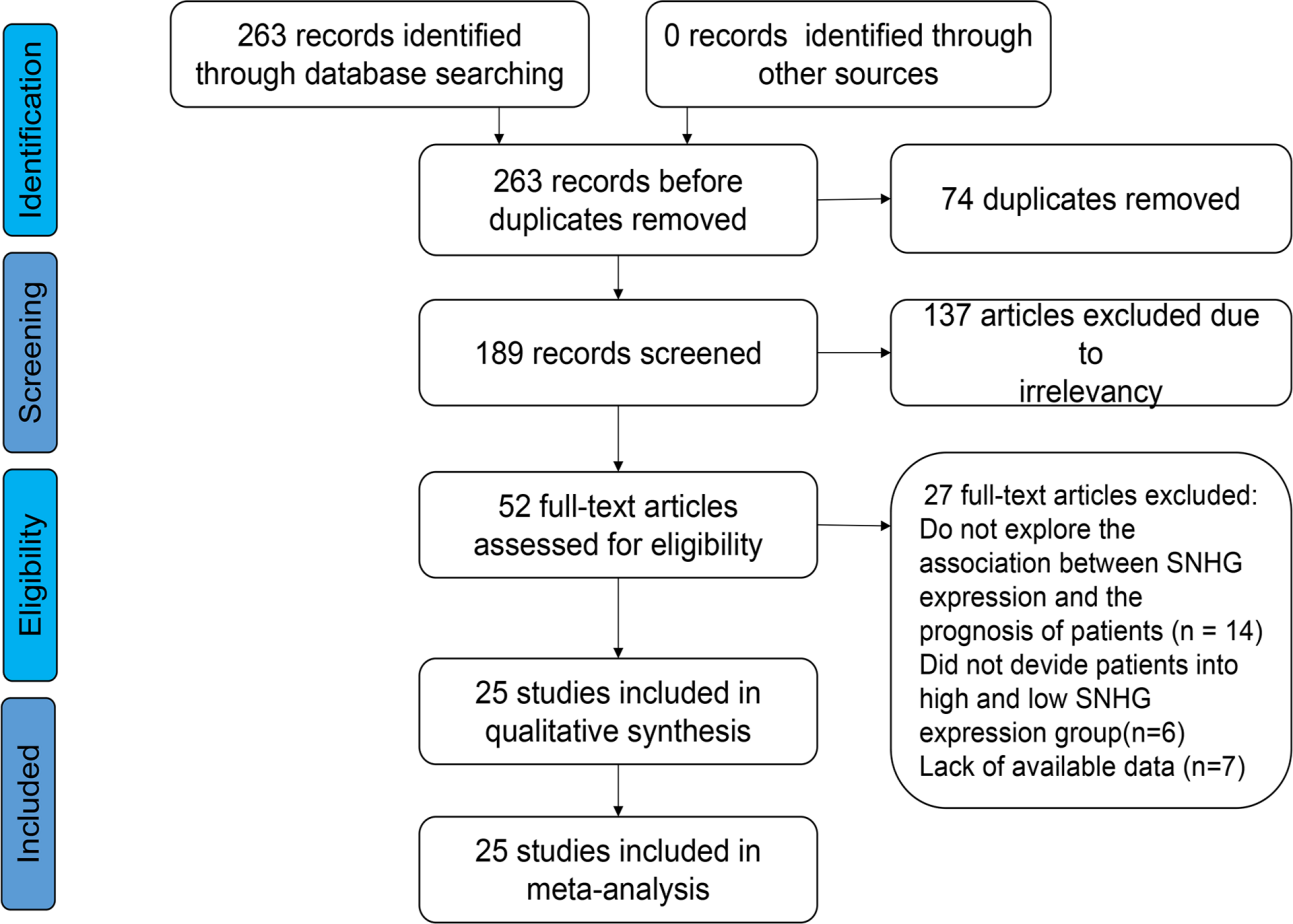
Flow diagram of the eligible studies.

For inclusion in this meta-analysis, two researches independently scored the selected studies using the Newcastle-Ottawa quality assessment scale (NOS) to assess the quality and suitability of the selected studies (http://www.ohri.ca/programs/clinical_epidemiology/oxford.asp) (16). The NOS items for selection of cohorts, comparability among included studies, and outcome were included. Study selection flow diagram is shown in **Figure 1**.

### 2.4 Data extraction

For all included literatures, two researchers independently extracted the following index in detail: name of first author, article release year, country where the patient belongs, number of cases, expression level of SNHG, cutoff value, presentation of HR value, follow-up month, HR with 95% CI for overall survival (OS) and reference-free survival (RFS); OR with 95% CI for dichotomous data included TNM staging, distant lymph node metastasis (LNM), organ metastasis, pathological score, tumor size etc. If the literature provides both multivariate and univariate analyses methods, then we extract the values of multivariate analysis. In situations where an article simply presents survival curves without providing HR and its 95% CI, the HR was extracted by extracting survival curves using the Engauge Digitizer v10.4 software (17). In the event where the extracted data of the two investigators were inconsistent, a third investigator was asked to evaluate the data to decide whether to extract the data or not. Additionally, we seek out The Cancer Genome Atlas (TCGA) database of Interactive Analysis of Gene Expression Profile, and explored the GEPIA2 website to further explore SNHG expression in colorectal tumor tissues and adjacent tissues.

### 2.5 Statistical analysis

Review Manager V 5.4 Software and Stata SE14.0 software were utilized to implement the statistical analysis in this study. patientss included in the study were spanided into high-expression and low-expression groups on the ground of reports in the original literature. Pooling OR besides 95% CI to investigate the correlation both lncSNHGs expression and the clinical prognostic parameters of CRC patientss, such as TNM stage, LNM, DM, histological grade, tumor size, depth of invasion, etc. Pooling HR with 95% CI was implemented to explore the pertinence of lncSNHGs expression levels with OS progression-free survival (PFS) and DFS in cancer patients. The heterogeneity amid incorporated studies was appraised by *I^2^* and P value. If *I^2^* was >50% and P<0.05, we assumed there exist markedly strengthened heterogeneity in this outcome and a random-effects model was performed. At the same time, we tried to found the provenience of heterogeneity through classification analysis in view of follow-up visits, cut-off index, HR calculation procedures, and sample size. Sensitivity analyses were performed to find individual studies with large heterogeneity in OS outcomes. Clinical and statistical bias was detected using Begg's test.

## 3 Results

### 3.1 Basic characteristics of included documents

Basic characteristics of enrolled documents according to our search using the keywords, 263 articles were screened in the initial trial, excluding 74 articles that were repeatedly published. One-hundred and thirty-seven papers did not assess the interrelation between lncRNA SNHGs expression and CRC prognosis, 14 papers were based on animal experiments, 6 papers did not classify lncRNA SNHGs into high and low expression patients, and 7 papers had insufficient data. This study finally included 25 suitable papers, including 2,342 CRC cases published between 2016 and 2022, with the number of patients ranging between 30–338 (14, 18–31). All the patients were from China, and were diagnosed as CRC by pathology or histology. High and low lncRNA SNHG expression was verified using the real-time quantitative reverse transcription reaction. Twenty-two papers included survival data of patients, and three papers only provided clinical pathological characteristics of patients (**Table 1**). The NOS score among covered investigations ranged between 6–9 based on overall evaluation of literature quality in detail (**Table 2**).

**Table 1:** Basic features of the publications included in this meta-analysis (n=25).

study/year	country	No. of patients	detection method	cut-off value	SNHG expression	survival analysis	HR statistics	hazard ratios (95%CI)	Analysis method	follow-up (month)	NOS score
Yao JN 2021	China	93	qRT-PCR	median	Up-regulated	OS	paper	1.45 (1.02-2.06)	Multivariate analysis	60	9
						DFS	paper	1.68 (1.04-2.71)	Multivariate analysis	60	9
Li C 2016	China	107	qRT-PCR	mean	Up-regulated	OS	paper	1.63 (1.22-3.98)	Multivariate analysis	36	8
Li YL 2019	China	56	qRT-PCR	median	Up-regulated	OS	survival curve	1.74 (0.72-4.2)	Univariate analysis	48	7
Pei Q 2019	China	32	qRT-PCR	mean	Up-regulated	OS	survival curve	2.04 (0.48-8.69)	Univariate analysis	60	7
Liu YH 2019	China	53	qRT-PCR	mean	Up-regulated	OS	survival curve	2.57 (1.12-5.90)	Univariate analysis	60	8
Wang JZ 2017	China	60	qRT-PCR	median	Up-regulated	OS	survival curve	2.70 (1.28-5.70)	Univariate analysis	72	8
Zhang PX 2020	China	96	qRT-PCR	median	Up-regulated	OS	paper	1.344 (1.058-1.709)	Multivariate analysis	36	9
Shan YJ 2018	China	48	qRT-PCR	median	Up-regulated	OS	survival curve	2.55 (0.87-7.50)	Univariate analysis	66	9
Chen Y 2019	China	141	qRT-PCR	mean	Up-regulated	OS	paper	2.83 (1.2-8.36)	Multivariate analysis	54	9
						RFS	paper	2.70 (1.17-6.20)	Multivariate analysis	54	9
Zhu YK 2018	China	40	qRT-PCR	mean	Up-regulated	OS	survival curve	2.61 (1.03-6.62)	Univariate analysis	60	8
Li M 2017	China	74	qRT-PCR	median	Up-regulated	OS	paper	2.568 (1.055-6.252)	Multivariate analysis	80	9
Li ZM 2018	China	66	qRT-PCR	median	Up-regulated	OS	survival curve	2.32 (1.00-5.41)	Univariate analysis	120	8
Zhang Y 2021	China	150	qRT-PCR	Not reported	Up-regulated	OS	survival curve	2.25 (1.41-3.58)	Univariate analysis	5	6
Wen DC 2020	China	58	qRT-PCR	mean	Up-regulated	OS	survival curve	1.83 (0.88-3.79)	Univariate analysis	60	8
Xu M 2018	China	130	qRT-PCR	mean	Up-regulated	OS	paper	2.82 (1.53–5.18)	Multivariate analysis	72	9
Xiang ZX 2022	China	111	qRT-PCR	median	Up-regulated	OS	paper	3.125 (1.145-8.474)	Multivariate analysis	60	9
						PFS	paper	1.869 (0.788-4.424)	Multivariate analysis	60	9
Huang L 2018	China	91	qRT-PCR	mean	Up-regulated	OS	paper	2.731 (1.005-7.424)	Multivariate analysis	72	9
Xu M 2019	China	120	qRT-PCR	mean	Up-regulated	OS	paper	2.48 (1.60–5.86)	Multivariate analysis	72	9
Fu Y 2019	China	80	qRT-PCR	mean	Up-regulated	OS	survival curve	2.41 (1.31-4.42)	Univariate analysis	60	8
						DFS	survival curve	2.19 (1.17-4.12)	Univariate analysis	60	8
Zhu YP 2017	China	108	qRT-PCR	median	Up-regulated	OS	paper	3.172 (1.554-6.209)	Multivariate analysis	60	9
						PFS	paper	2.893 (1.362-4.702)	Multivariate analysis	60	9
Tian T 2017	China	82	qRT-PCR	median	Up-regulated	OS	paper	1.45 (0.38-5.53)	Univariate analysis	120	8
						PFS	paper	2.23 (0.93-5.35)	Univariate analysis	120	8
Bai JH 2019	China	338	qRT-PCR	median	Up-regulated	OS	paper	2.222 (1.388-3.571)	Multivariate analysis	60	9
Lai FF 2020	China	40	qRT-PCR	mean	Up-regulated	not reported	–	–	–	–	6
Wang XY 2021	China	30	qRT-PCR	mean	Up-regulated	not reported	–	–	–	–	6
Zhou N 2021	China	70	qRT-PCR	mean	Up-regulated	not reported	–	–	–	–	6

SNHG, Small nucleolar RNA host genes; No., number; NA, not available; qRT-PCR, quantitative reverse transcription-polymerase chain reaction; paper, HR was extracted directly from article; survival curve: HR was extracted by extracting survival curves using the Engauge software; mean: The mean value was used as the cut-off value; median: The median value was used as the cut-off value; not reported: lack of survival data; OS, overall survival; DFS, disease free survival; RFS, recurrence free survival.

**Table 2:** Quality assessment of eligible studies (Newcastle-Ottawa scale) (NOS score).

Author (Reference)	Country	Selection				Comparability	Outcome			Total
		Adequate of case definition	Representativeness of the cases	Selection of Controls	Definition of Controls	Comparability of cases and controls	Ascertainment of exposure	Same method of ascertainment	Non-Response rate	
Yao JN 2021	China	*	*	*	*	**	*	*	*	9
Li C 2016	China	*	*	*	*	*	*	*	*	8
Li YL 2019	China	*	*	*	*	*	*	–	*	7
Pei Q 2019	China	–	*	*	*	*	*	*	*	7
Liu YH 2019	China	*	*	*	*	*	*	*	*	8
Wang JZ 2017	China	*	*	*	*	*	*	*	*	8
Zhang PX 2020	China	*	*	*	*	**	*	*	*	9
Shan YJ 2018	China	*	*	*	*	**	*	*	*	9
Chen Y 2019	China	*	*	*	*	**	*	*	*	9
Zhu YK 2018	China	–	*	*	*	**	*	*	*	8
Li M 2017	China	*	*	*	*	**	*	*	*	9
Li ZM 2018	China	*	*	*	*	*	*	*	*	8
Zhang Y 2021	Iran	*	*	*	*	*	*	NA	NA	6
Wen DC 2020	China	*	*	*	*	*	*	*	*	8
Xu M 2018	China	*	*	*	*	**	*	*	*	9
Xiang ZX 2022	China	*	*	*	*	**	*	*	*	9
Huang L 2018	China	*	*	*	*	**	*	*	*	9
Xu M 2019	China	*	*	*	*	**	*	*	*	9
Fu Y 2019	China	*	*	*	*	*	*	*	*	8
Zhu YP 2017	China	*	*	*	*	**	*	*	*	9
Tian T 2017	China	*	*	*	*	*	*	*	*	8
Bai JH 2019	China	*	*	*	*	**	*	*	*	9
Lai FF 2020	China	*	*	*	*	*	*	NA	NA	6
Wang XY 2021	China	*	*	*	*	*	*	NA	NA	6
Zhou N 2021	China	*	*	*	*	*	*	NA	NA	6

Reasons:

1. Insufficient sample size, resulting in statistical bias in research results (Li YL, 2019; Zhu YK, 2018).

2. The results were not analyzed in detail (without providing the data of multivariate analysis), resulting in statistical and artificial biases to some extent (Li C, 2016; Li YL, 2019; Pei Q, 2019; Liu YH, 2019; Wang JZ, 2017; Zhang PX, 2020; and Tian T, 2017) and lack of data on survival prognosis (Lai FF, 2020; Wang XY, 2021; Zhou N, 2021).

3. Lack of survival curve, unable to compare long-term prognosis of patients (NA).NOS uses the semi-quantitative principle of the star system to evaluate the quality of literature. Except for the maximum of 2 stars (**) for comparability, the other items can be evaluated by 1 star (*) with a full score of 9 stars. The higher the score, the higher the research quality.

### 3.2 Association between the expression level of SNHG and Survival Outcome of CRC Patients

**Figure 2:**
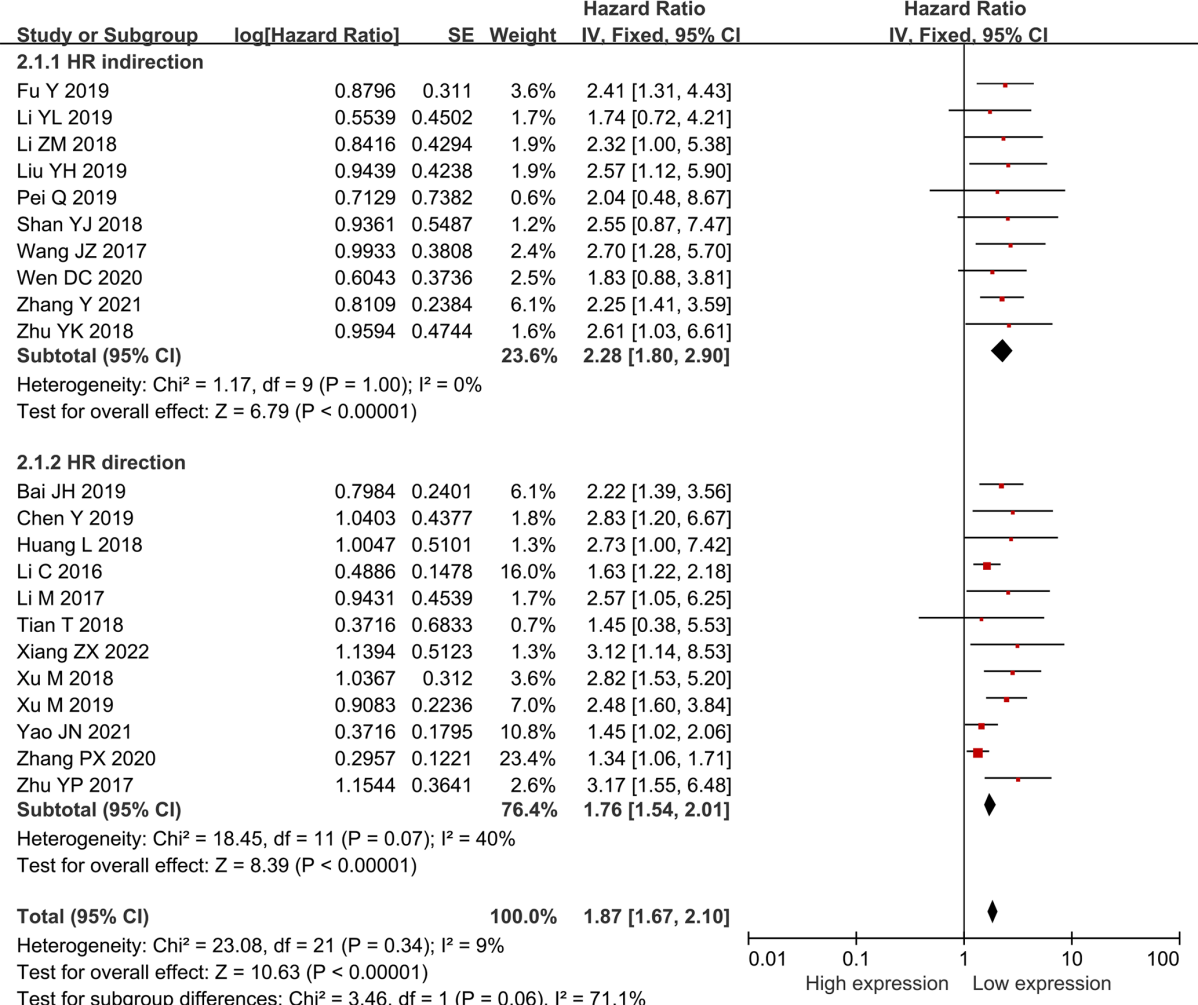
Forest plot showing the relationship between small nucleolar RNA host gene expression and overall survival (OS) in colorectal cancers.

**Figure 3:**
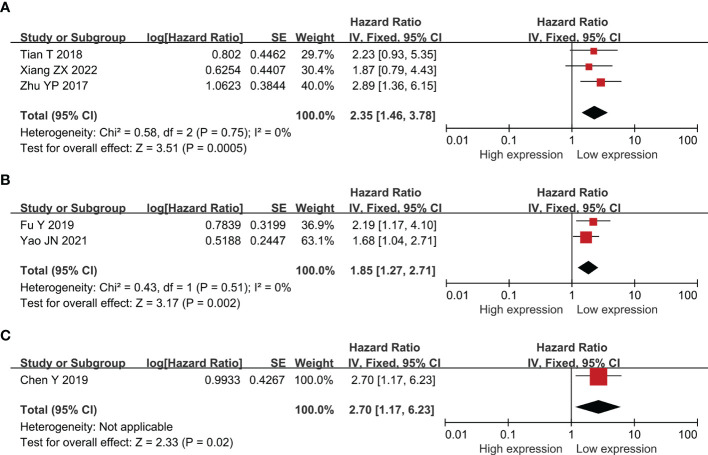
Forest plot showing the relationship between small nucleolar RNA host gene (SNHG) expression and progression-free survival (PFS) **(A)**; disease-free survival (DFS) **(B)**; and reference-free survival (RFS) in colorectal cancers **(C)**.

Twenty-two researches comprising 2,134 patientss were obtained to revealed the relativity between SNHG expression and the survival outcome of CRC patients. Twelve papers directly reported HR values and 95% CI; 10 papers simply provided survival curves and were extracted via the Engauge Software (indirect extraction). Pooled HRs demonstrated that high SNHG expression predicts poor cancer prognosis (HR = 1.635; 95% CI, 1.405–1.864; P<0.0001) (**Figure 2**). We took into consideration different cut-off index (mean or median), follow-up month, and HR estimation method (Directly and Indirectly). The classification analysis was carried out to reveal the marked heterogeneity between different groups based on HR estimation method (Directly and Indirectly), follow-up month (<60 months and not less than 60 months), number of patients (>100 and <100), cut-off value (mean and median), NOS score (9 and <9), and analysis method (univariate and multivariate analyses). A positive association was expose between increasing SNHG expression and short OS in the multivariate analysis (HR = 1.527; 95% CI, 1.276–1.779), univariate analysis (HR = 2.186; 95% CI, 1.616–2.756), HRs withdraw straight from studies (HR = 1.527; 95% CI, 1.276–1.777), or explicitly abstracted from the survival curve (HR = 2.224; 95% CI, 1.639–2.808), cancer with >100 (HR = 2.300; 95% CI, 1.729–2.871) and <100 (HR = 1.505; 95% CI, 1.254–1.757), not less than 60 months of follow-up (HR = 1.907; 95% CI, 1.546–2.267) and <60 months (HR = 1.448; 95% CI, 1.149–1.746) (**Table 3**). Three studies comprising 301 patients, 2 studies comprising 173 patientss, and 1 study with 141 patientss were obtained to evaluate the connection between SNHG expression and cancer survival outcome, including PFS, DFS, and recurrence free survival (RFS). Pooled HRs showed that increasing SNHG expression reminds worse PFS (HR = 2.35; 95% CI, 1.46–3.78) (**Figure 3A**), DFS (HR = 1.85; 95% CI, 1.27–2.71) (**Figure 3B**), and RFS (HR = 2.70; 95% CI, 1.17–6.23) (**Figure 3C**).

**Table 3:** Subgroup analysis of lncRNA SNHGs expression and overall survival in colorectal cancer patients.

		No. of studies		No. of patients		Pooled HR (95% CI)		Heterogeneity	
						Fixed	Random	*I* ^2^(%)	*P-*value
**Overall survival**		22		1955		1.870 (1.670-2.10)	1.940 (1.710-2.210)	9	0.34
Analysis method									
Univariate analysis		11		725		2.186 (1.616-2.756)	2.186 (1.616-2.756)	0	0.999
Multivariate analysis		11		1230		1.527 (1.276-1.779)	1.527 (1.276-1.779)	0	0.449
HR estimation method									
Indirectly		10		643		2.224 (1.639-2.808)	2.224 (1.639-2.808)	0	0.999
Directly		12		1312		1.527 (1.276-1.777)	1.527 (1.276-1.777)	0	0.539
Cut-off value									
Median		11		953		1.507 (1.254-1.760)	1.507 (1.254-1.760)	0	0.618
Mean		10		852		2.207 (1.554-2.861)	2.207 (1.554-2.861)	0	0.986
Not reported		1		150		2.250 (1.410-3.580)	2.250 (1.410-3.580)	–	–
number of patients									
more than 100		8		1026		2.300 (1.729-2.871)	2.300 (1.729-2.871)	0	0.955
less than 100		14		929		1.505 (1.254-1.757)	1.505 (1.254-1.757)	0	0.89
Follow-up (month)									
Not less than 60 months		17		1405		1.907 (1.546-2.267)	1.907 (1.546-2.267)	0	0.928
Less than 60 months		5		550		1.448 (1.149-1.746)	1.448 (1.149-1.746)	0	0.519
Quality scores									
Score = 9		11		1171		1.530 (1.275-1.785)	1.560 (1.282-1.838)	2.4	0.419
Score < 9		11		784		2.093 (1.560-2.627)	2.093 (1.560-2.627)	0	0.998
**PFS**		3		301		2.350 (1.460-3.780)	2.350 (1.460-3.780)	0	0.75
**DFS**		2		173		1.850 (1.270-2.710)	1.850 (1.270-2.710)	0	0.51
**RFS**		1		141		2.70 (1.170-6.230)	2.70 (1.170-6.230)	–	–

SNHG, Small nucleolar RNA host genes; OS, overall survival; DFS, disease-free survival; RFS, recurrence free survival; PFS, progression-free survival; Random, Random effects; and Fixed, Fixed effects; Directly: Hazards ration (HR) was extracted directly from the primary articles; and indirectly: HR was extracted indirectly from the primary articles.

### 3.3 Association between the expression level of SNHG and TNM stage

**Figure 4:**
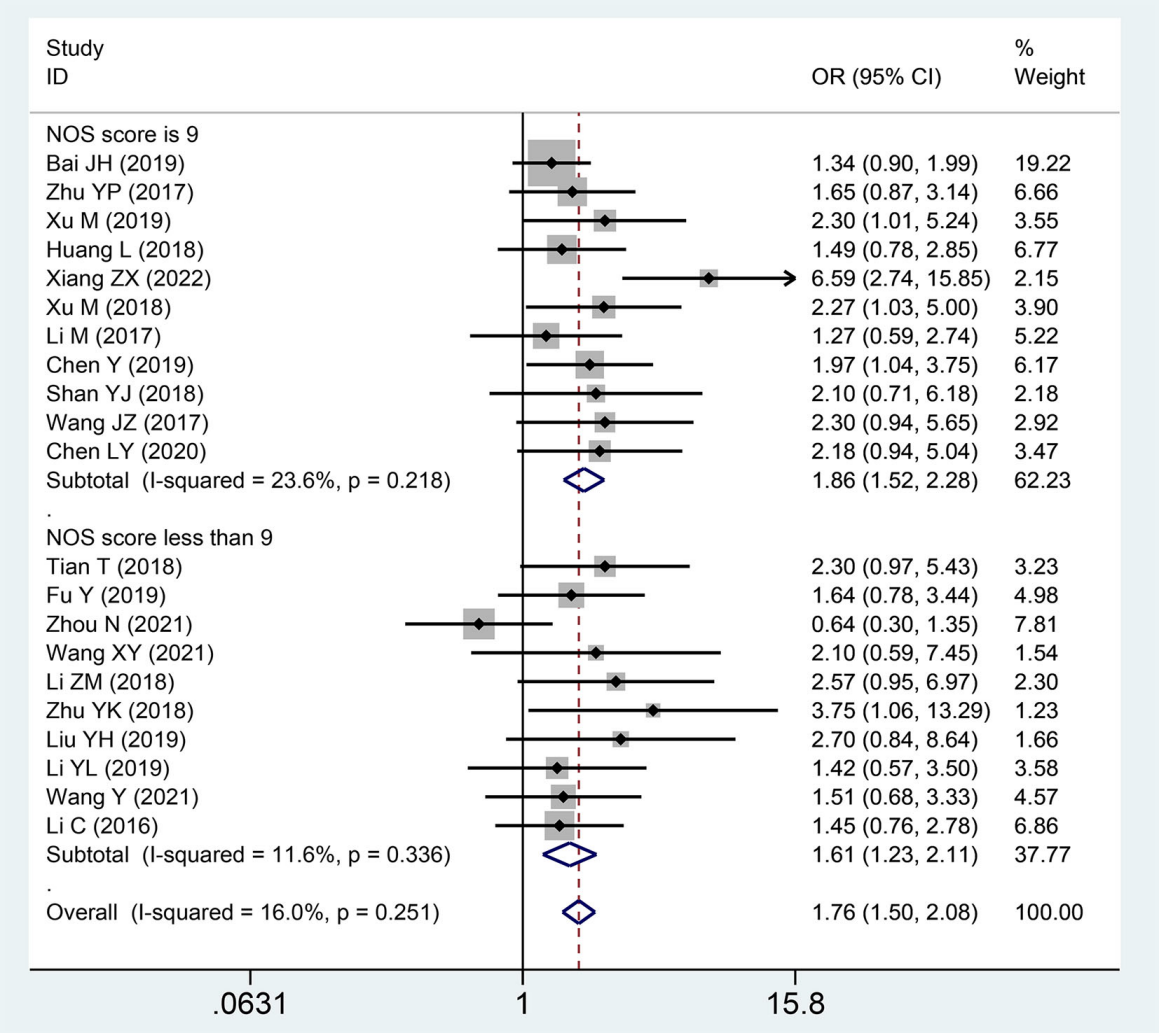
Forest plot showing the relationship between small nucleolar RNA host gene (SNHG) expression and TNM stage in colorectal cancers.

Twenty publications with 1,873 patientss were obtained to inquire the dependency between lncRNA SNHGs expression and clinical stage of CRC patientss. The publications showed that increasing SNHG expression predicts an poor TNM stage (OR = 1.750; 95% CI, 1.481–2.067; P=0.0001) (**Figure 4**). A classification analysis redouble revealed that high SNHG expression means a terminal TNM stage in a group that used mean value as the cut-off value (OR = 1.656; 95% CI, 1.315–2.085; P=0.0001) and median value as the cut-off value (OR = 1.859; 95% CI, 1.460–2.368; P=0.0001). At the same time, enhanced SNHG expression indicates advanced TNM stage in both high NOS score (OR = 1.841; 95% CI, 1.490–2.274; P=0.0001) and low (OR = 1.609; 95% CI, 1.226–2.110; P=0.0001) NOS score groups. A fixed effects model was exerted to derive from a small heterogeneity level (*I^2^* = 19.2, P=0.216) (**Table 4**).

**Table 4:** Pool effects of clinicopathological characteristics in colorectal cancer patients with abnormal lncRNA SNHGs expression.

Clinicopathologic characteristics	No. of studies	No. of patients	Odds ratio (95% CI)		*P*	Heterogeneity	
			Fixed	Random		*I* ^2^(%)	*P*-value
**Age**	24	2141	1.055 (0.916-1.216)	1.055 (0.916-1.216)	0.456	0	1
**gender**	23	2079	0.951 (0.817-1.107)	0.951 (0.817-1.107)	0.518	0	1
**TNM (**III+IV vs. I+II)	21	1953	1.765 (1.499-2.078)	1.765 (1.499-2.078)	0.0001	16	0.251
cut-off value							
median	9	969	1.882 (1.492-2.375)	1.981 (1.447-2.711)	0.0001	35.5	0.134
mean	12	984	1.656 (1.315-2.085)	1.648 (1.304-2.082)	0.0001	0.3	0.251
NOS score							
9	11	1301	1.860 (1.515-2.283)	1.897 (1.482-2.430)	0.001	23.6	0.218
less than 9	10	652	1.609 (1.226-2.110)	1.619 (1.203-2.179)	0.001	11.6	0.336
**LNM (present vs. absent)**	17	1389	1.645 (1.338-2.021)	2.03(1.08-3.83)	0.028	82.2	0
cut-off value							
median	4	343	2.073 (1.393-3.086)	2.184 (1.190-4.009)	0.012	35.5	0.134
mean	13	1046	1.507 (1.184-1.919)	1.492 (1.168-1.907)	0.0001	0.3	0.251
NOS score							
9	8	845	1.793 (1.347-2.388)	1.770 (1.263-2.481)	0.001	21.1	0.262
less than 9	9	544	1.496 (1.111-2.014)	1.484 (1.094-2.012)	0.001	1.2	0.424
**DM (present vs. absent)**	18	1694	1.601 (1.299-1.973)	1.718 (1.225-2.410)	0.002	46.6	0.016
cut-off value							
median	5	699	1.381 (1.016-1.876)	1.435 (0.912-2.256)	0.118	27.5	0.238
mean	13	995	1.815 (1.363-2.418)	1.833 (1.158-2.901)	0.01	51.2	0.017
NOS score							
9	8	1087	1.604 (1.227-2.096)	1.919 (1.078-3.416)	0.001	64.7	0.006
less than 9	10	607	1.596 (1.143-2.229)	1.589 (1.045-2.416)	0.006	25	0.213
**Tumor size (big vs small)**	12	1158	1.297 (1.061-1.584)	1.290 (1.041-1.600)	0.02	6.9	0.378
cut-off value							
median	4	617	1.372 (1.046-1.800)	1.370 (1.033-1.818)	0.029	4	0.373
mean	8	541	1.212 (0.901-1.630)	1.187 (0.850-1.659)	0.314	16	0.304
NOS score							
9	7	880	1.450 (1.150-1.829)	1.442 (1.142-1.821)	0.002	0	0.451
less than 9	5	278	0.927 (0.621-1.383)	0.925 (0.617-1.387)	0.709	0	0.627
**Histological grade**	11	1159	1.416 (1.111-1.805)	1.414 (1.109-1.803)	0.005	0	0.995
cut-off value							
median	7	861	1.466 (1.115-1.926)	1.465 (1.114-1.926)	0.006	0	0.99
mean	4	298	1.245 (0.734-2.111)	1.236 (0.727-2.103)	0.416	0	0.805
NOS score							
9	7	878	1.415 (1.073-1.866)	1.414 (1.072-1.865)	0.014	0	0.986
less than 9	4	281	1.420 (0.857-2.353)	1.412 (0.849-2.349)	0.173	0	0.757
**Invasion depth (T3+T4/T1+T2)**	7	653	1.603 (1.218-2.110)	1.590 (1.204-2.098)	0.001	0	0.483

SNHG, small nucleolar RNA host genes; LNM, lymph node metastasis; Random, random-effect model; TNM, TNM stage; DM, distant metastasis; Fixed, Fixed effects model; NOS, Newcastle-Ottawa Scale; CI, Confidence interval.

### 3.4 Association between the expression level of SNHG and lymph node metastasis

**Figure 5:**
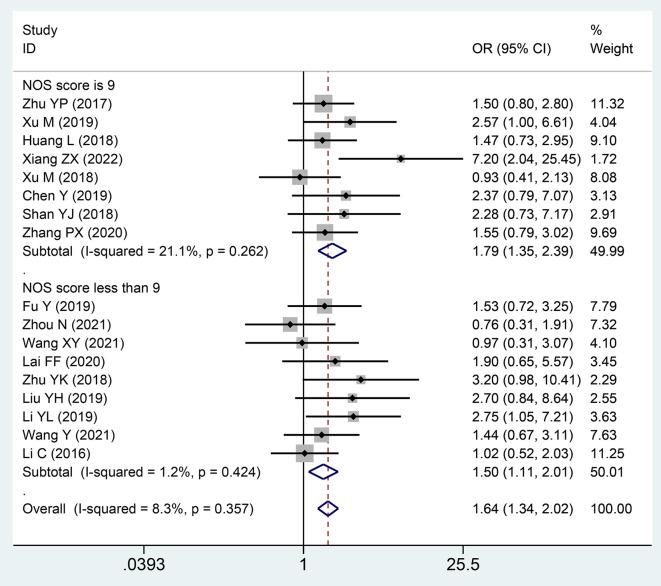
Forest plot showing the relationship between small nucleolar RNA host gene (SNHG) expression and lymph node metastasis (LNM) stage in colorectal cancers.

Seventeen publications with 1,389 patientss were incorporated to inspect the pertinence between the lncRNA SNHGs expression and LNM of CRC patientss. These studies showed that high SNHG expression was noteworthy relativity to lymph node invasion (OR = 1.645; 95% CI, 1.338–2.021; P=0.0001) (**Figure 5**). In a subgroup analysis, we detected that elevated lncRNA SNHGs expression demonstrated significant association with lymph node metastasis in a group that used mean value as the cut-off index (OR = 1.507; 95% CI, 1.184–1.919; P=0.0001) and median value as the cut-off index (OR = 2.073; 95% CI, 1.393–3.086; P=0.0001). A remarkable relevance was observed in both high (OR = 1.793; 95% CI, 1.347–2.388; P=0.0001) and low (OR = 1.496; 95% CI, 1.111–2.014; P=0.0001) NOS score groups. A fixed effects model was adopted for small heterogeneity level (*I^2^* = 8.3, P=0.357) (**Table 4**).

### 3.5 Association between the expression level of SNHG and DM

**Figure 6:**
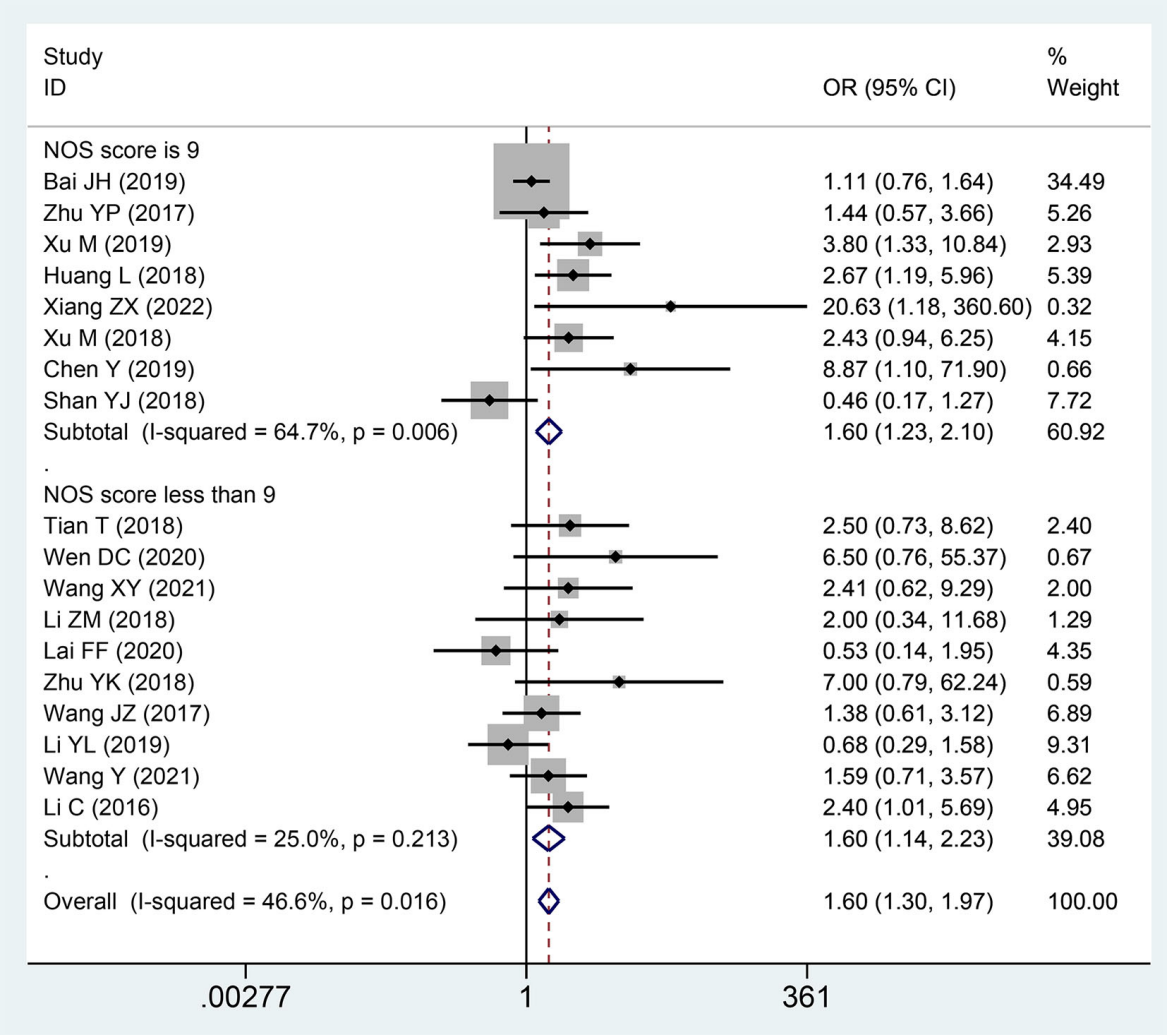
Forest plot showing the relationship between small nucleolar RNA host gene (SNHG) expression and distant metastasis (DM) stage in colorectal cancers.

A total of 18 studies with 1,694 patients described the association between the correlation of the expression level of lncRNA SNHGs and distant metastasis in patients with CRC. A significantly positive correlation was revealed between increased lncRNA SNHGs expression and earlier distant metastases (OR = 1.601; 95% CI, 1.299–1.973; *P*=0.0001) (**Figure 6**). A subgroup analysis further found a positive association between elevated SNHG expression and earlier distant metastases in the group that used mean value as the cut-off value (OR = 1.815; 95% CI, 1.363–2.418; *P*=0.0001) and median value as the cut-off value (OR = 1.381; 95% CI, 1.016–1.876; *P*=0.0001). A similar positive relationship was observed in groups with high (OR = 1.604; 95% CI, 1.227–2.096; *P*=0.0001) and low (OR = 1.596; 95% CI, 1.143–2.229; *P*=0.0001) NOS scores. A fixed effects model was adopted due to small heterogeneity level (*I^2^* = 46.6, *P*=0.016) (**Table 4**).

### 3.6 Association between the expression level of SNHG and Tumor Size

**Figure 7:**
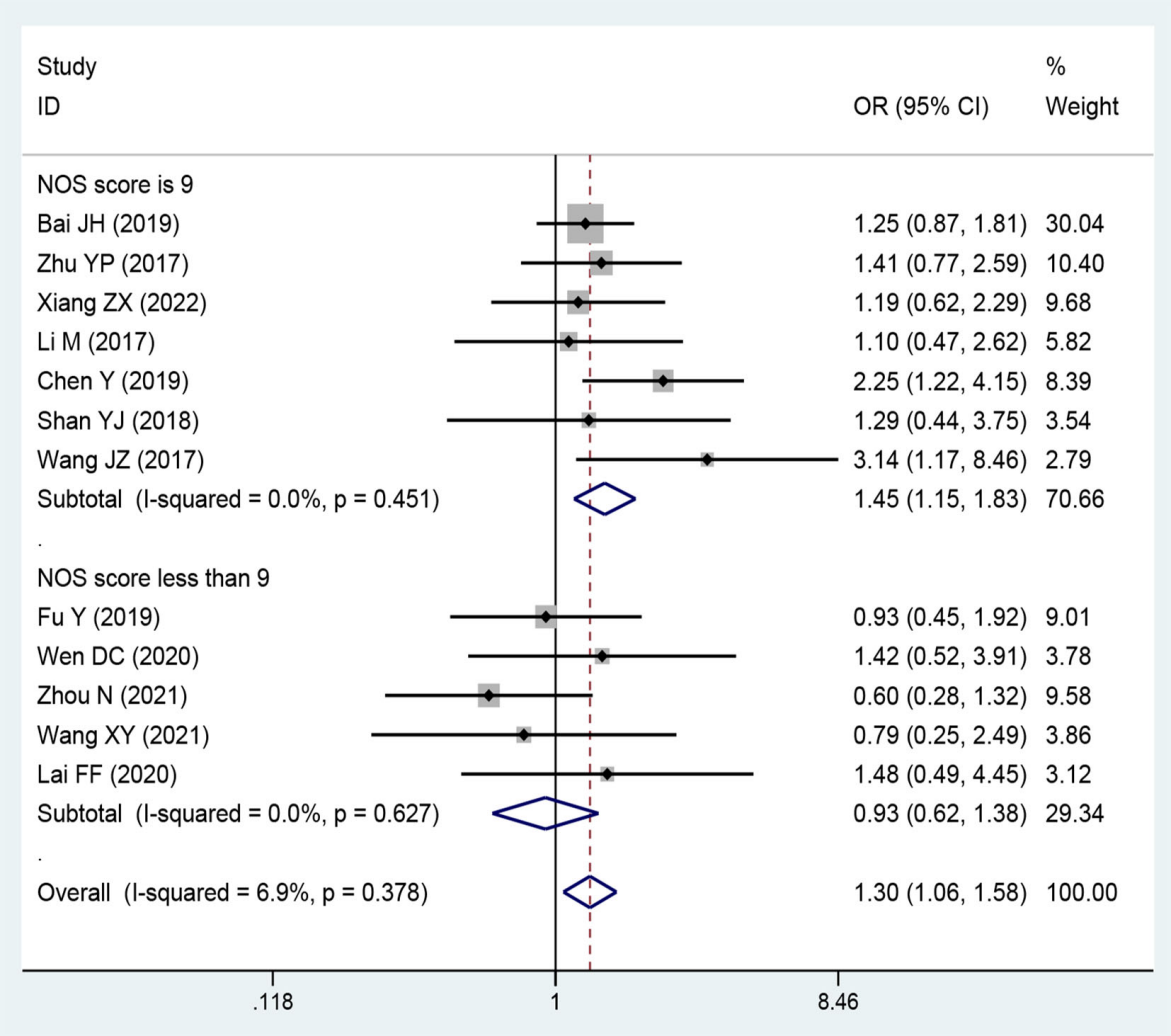
Forest plot showing the relationship between small nucleolar RNA host gene (SNHG) expression and tumor size stage in colorectal cancers.

Twelve publications comprising 1,158 patients were included to assess the relationship between the expression level of lncRNA SNHGs and tumor size in CRC patients. A positive association was observed between high lncRNA SNHGs expression and bigger tumor size (OR = 1.297; 95% CI, 1.061–1.584; *P*=0.011) (**Figure 7**). A subgroup analysis was based on a cut-off value and NOS score, and significantly positive correlations were also confirmed in groups that used median value as the cut-off value (OR = 1.372; 95% CI, 1.046–1.800; *P*=0.0001) and those with high NOS score (OR = 1.450; 95% CI, 1.150–1.829; *P*=0.0001). Meanwhile, no significance was observed in groups that used mean value as the cut-off value (OR = 1.212; 95% CI, 0.901–1.630; *P*=0.0001) and those with low NOS score (OR = 0.927; 95% CI, 0621–1.383; *P*=0.0001). A fixed effects model was adopted due to small heterogeneity level (*I^2^* = 46.6, *P*=0.016) (**Table 4**).

### 3.7 Association between the expression level of SNHG and other clinicopathological indicators

The relationship between lncRNA SNHGs expression and other clinicopathological indicators, including histological grade, age, and gender were also assessed. A significantly positive correlation was observed between high SNHG expression and depth of invasion (HR = 1.603; 95% CI, 1.218–2.110; *P*=0.001) and bad histological grade (HR = 1.416; 95% CI, 1.111–1.805; *P*=0.005). Meanwhile, no significance was observed between lncRNA SNHGs expression and age (OR = 1.055; 95% CI, 0.916–1.216; *P*=0.456) and gender (OR = 0.951; 95% CI, 0.817–1.107; *P*=0.61) (**Table 4**).

### 3.8 Sensitivity analysis and publication bias

**Figure 8:**
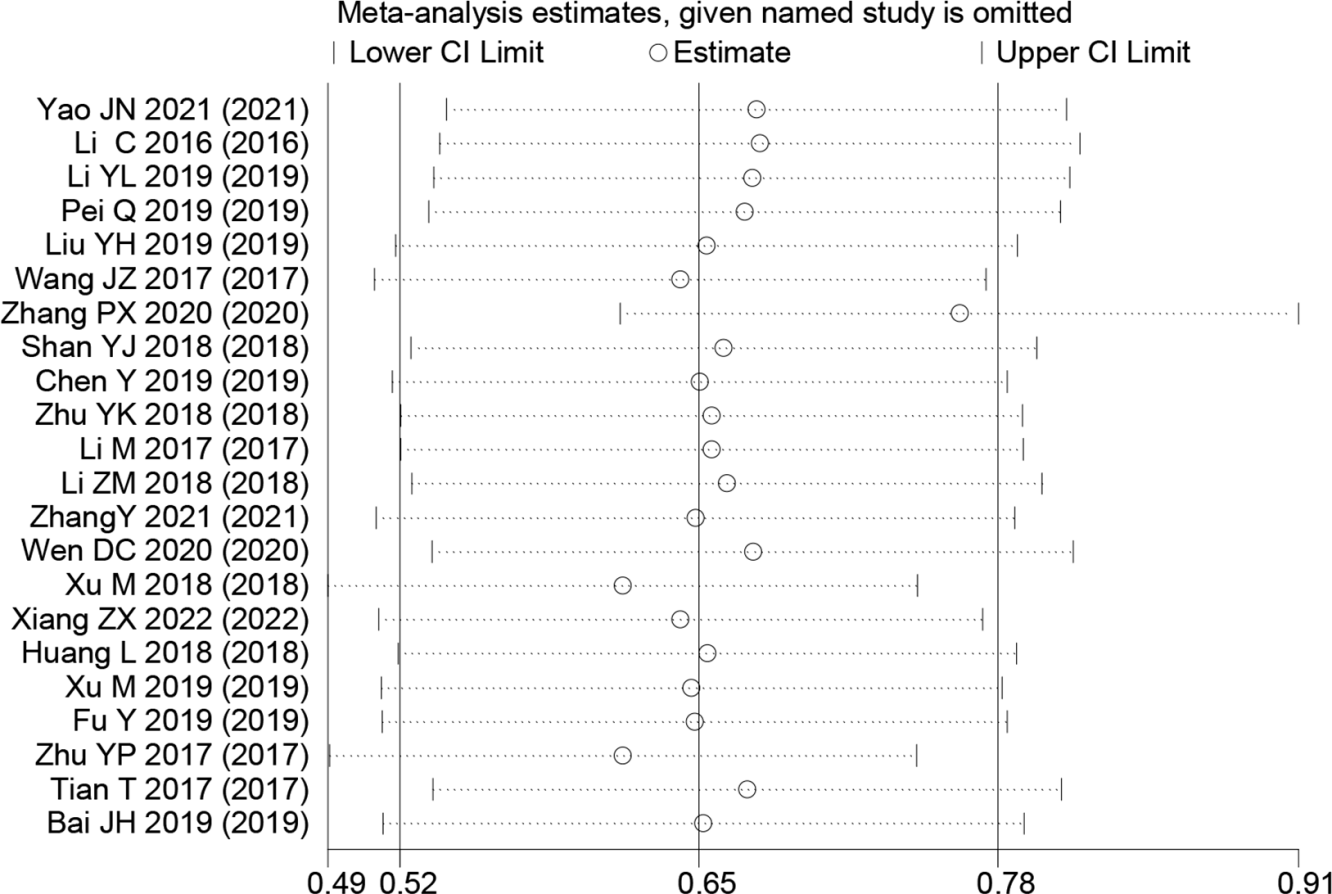
Sensitivity analysis for small nucleolar RNA host gene expression with overall survival in colorectal cancers. HR, hazard ratio; CI, confidence interval.

**Figure 9:**
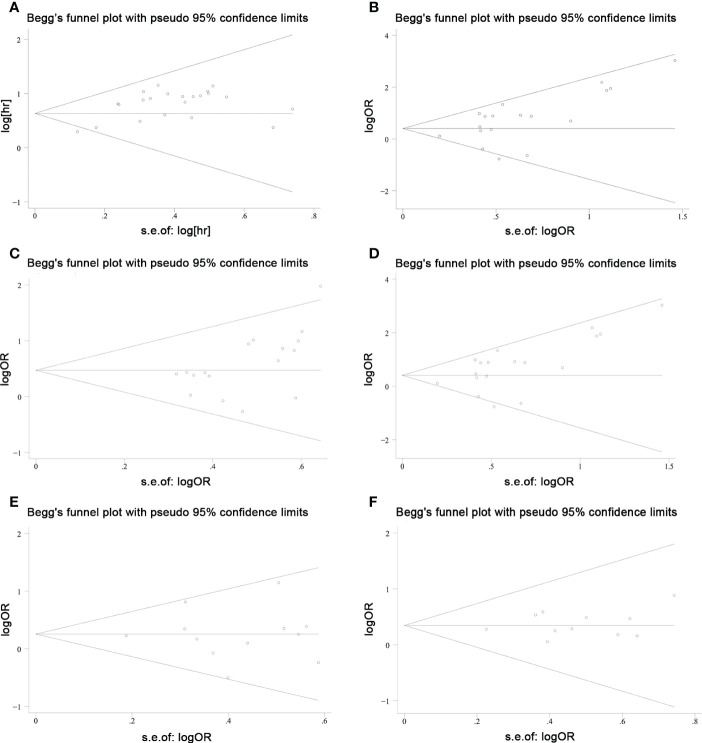
Publication bias was explored using Begg’s test in colorectal cancers. **(A)** overall survival; **(B)** TNM stage; **(C)** lymph node metastasis; **(D)** distant metastasis; **(E)** tumor size; and **(F)** histological grade.

Sensitivity analyses were performed to explore methodological heterogeneity in these studies, and to explore whether single study data significantly affected the overall outcome. We did not find a study data that affected the overall outcome significantly (**Figure 8**). A potential publication bias was explored using the Begg’s test, and unobvious publication bias was found in the included studies (**Figure 9**).

### 3.9 Bioinformatics analysis

**Figure 10:**
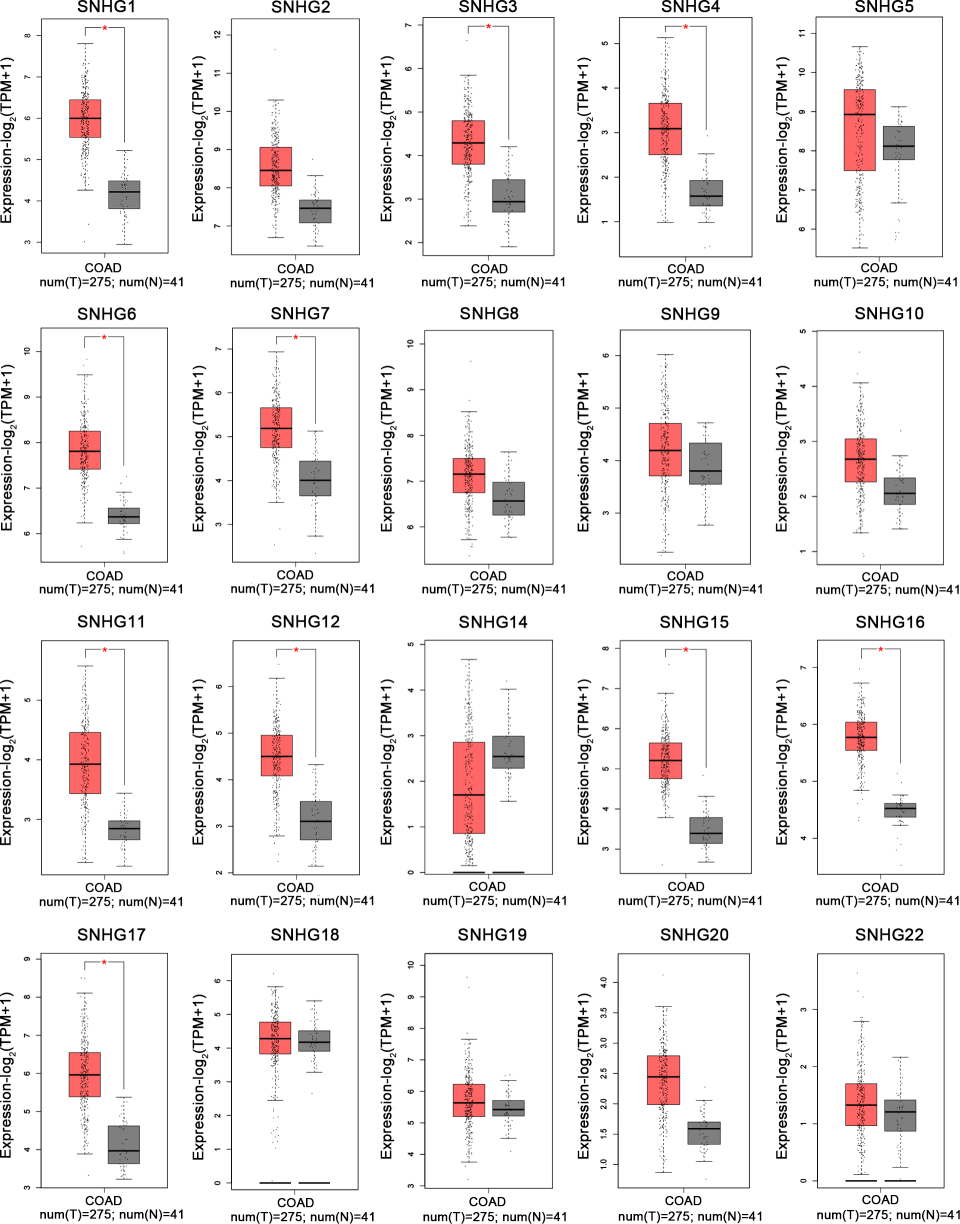
Expression levels of SNHG1-SNHG22 in colonic tumor and normal tissues in the GEPIA cohort by merging SNHG expression data (n=316).

Based on TCGA-COAD and TCGA-READ datasets of TCGA database (https://portal.gdc.cancer.gov/repository?facetTab=cases) and the GEPIA website (http://gepia.cancer-pku.cn/), we analyzed the expression of SNHGs in tumor tissues and adjacent tissues. The results showed that the expression level of SNHGs in tumor tissues was higher than that in adjacent tissues. This result is consistent with the results of this meta-analysis. (**Figure 10**).

## 4 Discussion

**Figure 11:**
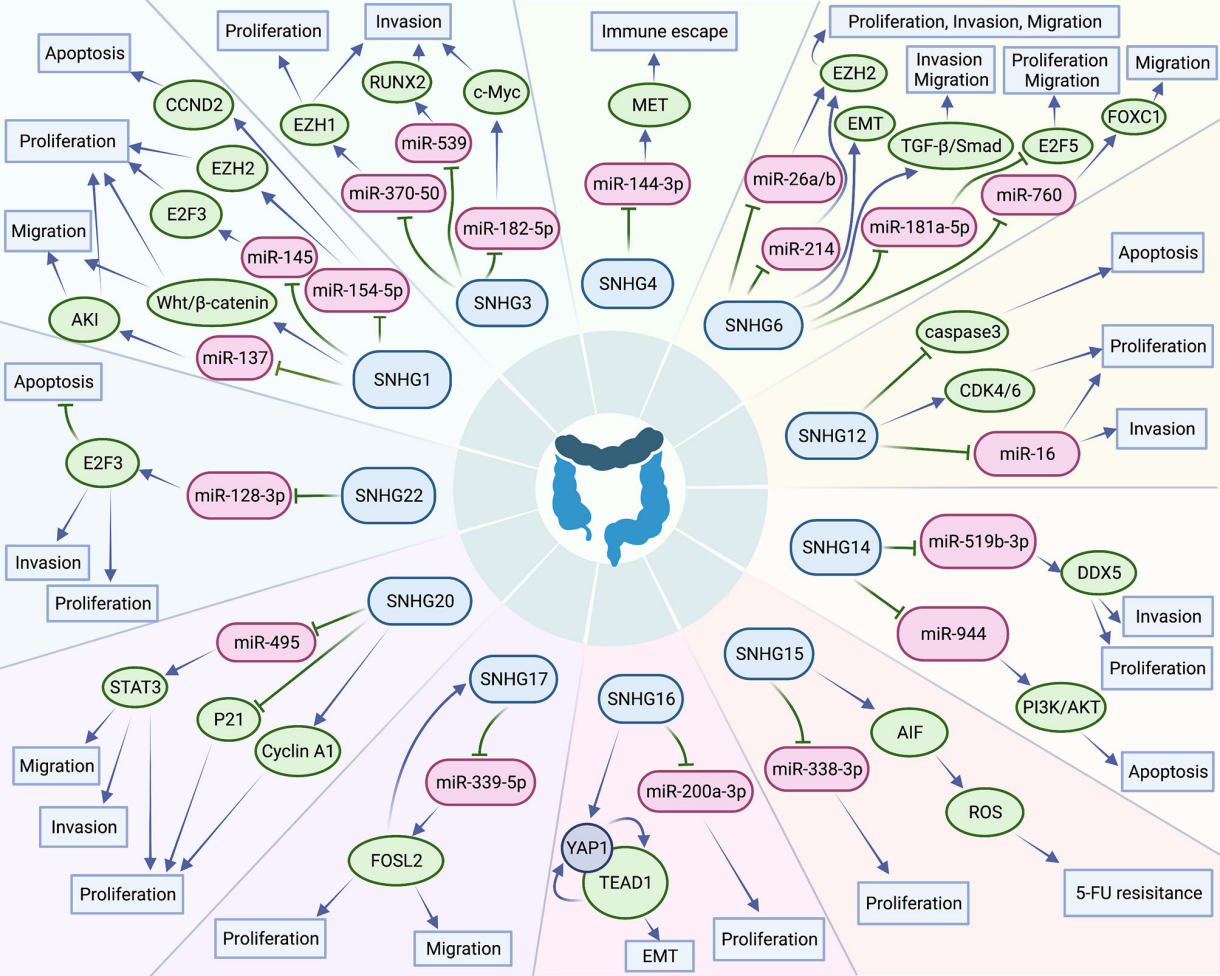
Small nucleolar RNA host genes involved in a series of cellular biological roles in colorectal cancers.

In the past ten years, increasing lncRNA SNHGs have been confirmed to regulate the cell biological behavior of CRC cells, for instance, both proliferation, chemotherapy resistance and immune escape (32). Many researchers tried to control the expression quantity of lncRNA SNHGs to achieve the impact of treating CRC. Mounting evidence attempts to seek the relativity between lncRNA SNHGs expression and survival outcome of CRC (33). It is gratifying that most studies have proclaimed that high expression of lncRNA SNHGs is memorably bound up positively with poor prognosis of CRC (23, 34). However, due to insufficient data in a single study and inconsistent research conclusions of several researches, so far, no research has systematically probed the relations between outlier expression of SNHG and CRC prognosis. In this paper, we summarized and explored the intervention of lncRNA SNHGs in colorectal malignancies. In this study, 25 original studies were included, and lncRNAs were highly expressed in CRC. This finding could also be obviously supported by TCGA and GEPIA (**Figure 10**). Pooling HR with 95% CI showed high expression of lncSNHG, thereby indicating poor cancer prognosis, OS, PFS, and DFS. We further explored the prognostic relevance of lncSNHGs through subgroup analysis. The results stated clearly that increased lncRNA SNHGs expression emphatically predicted unsatisfactory colorectal prognosis in various subgroup such as HR direct extraction group (Detail in **Table 3**). Additionally, some studies also tried to explore the correlation between lncRNA SNHGs expression and other prognostic indicators, including PFS, DFS, and RFS of CRC patients. Pooled HR with 95% CI make clear that high lncRNA SNHGs expression was positively interrelated with worse PFS, DFS and RFS. This study also seek the relativity between lncRNA SNHGs expression and clinical pathological parameters. The results showed that high lncRNA SNHGs predicted advanced clinical stage, deeper invasion, worse differentiation grade, and larger tumor size. Overall, lncRNA SNHGs was positively correlated with poor CRC prognosis, and lncRNA SNHGs may be an excellent indicator of survival prognosis and a aussichtsreich therapeutic target for CRC. Increasing researchers are attempting inquired the underlying biological mechanisms of lncRNA SNHGs in CRC cells at the molecular level (**Table 5** and **Figure 11**). First, SNHG could directly bind on downstream target proteins, thereby affecting the tumor cell growth, metastasis and apoptosis of through some signal cascades. For example, Huang et al. demonstrated that lncSNHG15 is preeminently associated with liver metastasis of CRC, but the specific mechanism has not yet been discovered (35). Li et al. reported that SNHG 20 could induce cell growth, distant metastasis and inhibit cell apoptosis of CRC cells by modulating P21 and cyclin A1 (19). Wang et al. reported that lncSNHG6 may drive the migration, growth and make inroads on CRC cells via TGF-β/Smad signaling pathway activation by targeting UPF1 (36). Shan et al. speculated that lncSNHG7 induces the migration and proliferation of CRC cells by enhanced N-acetylgalactosaminyltransferase 1 expression and promoting epithelial-mesenchymal transition (EMT) markers (E-cadherin and vimentin) by binding to miR-216b (25). Wang et al. revealed that lnSNHG12 promotes growth by upregulating cell cycle-related proteins and stifled apoptosis via decrease in caspase 3 expression (23).

**Table 5:** Transition of cell phenotype and related molecular mechanisms with abnormal lncRNA SNHGs expression in colorectal cancers.

lncRNA	Cancer type	Expression	Role	Micro-RNAs	Targets/signaling pathway	Functions	References
SNHG22	colorectal cancer	up-regulation	Oncogene	miR-128-3p	E2F3	promote proliferation, migration and invasion; inhibit apoptosis	Yao JN 2021
SNHG20	colorectal cancer	up-regulation	Oncogene		p21 cyclin A1	promote cell proliferation, invasion and migration, and cell cycle	Li C 2021
SNHG16	colorectal cancer	up-regulation	Oncogene	miR-200a-3p		promote proliferation, migration and invasion	Li YL 2019
SNHG14	colorectal cancer	up-regulation	Oncogene	miR-944	KRAS, PI3K/AKT	promote proliferation, migration, invasion and suppresses apoptosis	Pei Q 2019
SNHG12	colorectal cancer	up-regulation	Oncogene	miR-16		promote proliferation and invasion	Liu YH 2019
SNHG12	colorectal cancer	up-regulation	Oncogene		CDK4, CDK6, CCND1	promotes proliferation and inhibits apoptosis	Wang JZ 2017
SNHG7	colorectal cancer	up-regulation	Oncogene			promote proliferation and invasion	Zhang PL 2020
SNHG7	colorectal cancer	up-regulation	Oncogene	miR-216b	GALNT1, Bax, caspase-3	inhibit apoptosis	Shan YJ 2018
SNHG6	colorectal cancer	up-regulation	Oncogene	miR-181a-5p	SNHG6/miR-181a-5p/E2F5 axis	promote cell proliferation, migration and invasion, inhibit G0/G1 arrest and apoptosis	Yu C 2019
SNHG6	colorectal cancer	up-regulation	Oncogene	miR-760	SNHG6/miR-760/FOXC1	promote cell proliferation, invasion and migration	Zhu YK 2018
SNHG6	colorectal cancer	up-regulation	Oncogene	miR-181	JAK2	promotes proliferation and inhibits apoptosis	Lai FF 2020
SNHG6	colorectal cancer	up-regulation	Oncogene			promote cell proliferation, cell cycle progression, and inhibit apoptosis	Li M 2017
SNHG6	colorectal cancer	up-regulation	Oncogene		p21	promote cell proliferation	Li ZM 2018
SNHG14	colorectal cancer	up-regulation	Oncogene	miR-519b-3p	Bax/bcl-2/caspase9/caspase3	promotes cell proliferation and invasion	Wang XY 2021
SNHG4	colorectal cancer	up-regulation	Oncogene	miR-144-3p	miR-144-3p/MET	promote tumor cell immune escape	Zhou N 2021
SNHG3	colorectal cancer	up-regulation	Oncogene	miR-370-5p	miR-370-5p/EZH1	promote cell proliferation and invasion	Zhang Y 2021
SNHG3	colorectal cancer	up-regulation	Oncogene	miR-539	miR-539/RUNX2	promote proliferation and migration	Wen DC 2020
SNHG1	colorectal cancer	up-regulation	Oncogene	miR-154-5p	EZH2, CDKN2B, CCND2	promote cell proliferation	Xu M 2018
SNHG16	colorectal cancer	up-regulation	Oncogene	miR-195-5p	SNHG16-YAP1/TEAD1 positive feedback loop	promote cell proliferation and invasion	Xiang ZX 2022
SNHG12	colorectal cancer	up-regulation	Oncogene	microR-195		promote cell proliferation, migration and invasion	Chen LY 2020
SNHG6	colorectal cancer	up-regulation	Oncogene	miR-26a/b, miR-214	EZH2	promote cell proliferation, migration and invasion	Xu M 2019
SNHG1	colorectal cancer	up-regulation	Oncogene	miR-137	PI3K/AKT	promote cell proliferation and migration	Fu Y 2019
SNHG1	colorectal cancer	up-regulation	Oncogene		Wnt/β-catenin	promotes cell proliferation and metastasis	Zhu YP 2017
SNHG1	colorectal cancer	up-regulation	Oncogene	miR-145		promotes cell proliferation	Tian T 2018
SNHG1	colorectal cancer	up-regulation	Oncogene	miR-497/miR-195-5p	E-cadherin, N-cadherin, Vimentin	induce EMT	Bai JH 2019

SNHG, small nucleolar RNA host genes; GALNT1, N-acetylgalactosaminyltransferase 1; JAK2, janus kinase 2; CDKN2B, cyclin dependent kinase inhibitor 2B; CCND2, recombinant cyclin D2; E2F3, E2F transcription factor 3; ZEB1, zinc finger E-box binding homeobox 1; KRAS, kirsten rat sarcoma viral oncogene; YAP1, Yes-associated protein 1; MET, mesenchymal-epithelial transition factor; CDK4, cyclin-dependent kinase 4; CDK6, cyclin-dependent kinase 6; NA, not available.

MicroRNAs have been proved to play an critical part in the progress of CRC (37). Different expression of microRNAs could surpassingly promote growth, local invasion, and distant organ migration of CRC cells (38). Additionally, lncSNHGs could work as competitive endogenous RNA (ceRNA) (**Figure 10**, thereby influencing the biological characteristics of CRC tumor cells by affecting the downstream signal axis through sponge adsorption of microRNAs. Bai et al. indicated that lncSNHG1 may functions as an oncogene in order to drive CRC cell violation and transference via EMT modification by cooperating with miR-195-5p/miR-497, downregulating E-cadherin, and upregulating N-cadherin and vimentin (12). Zhang et al. uncovered that lncSNHG3 facilitates callus grow and violation of CRC cells by upregulating the enhancer of zeste homolog 1 (EZH1) and downregulating miR-375-5p (32). Similarly, Wen et al. revealed that lncSNHG3 induces the cell multiplication and migration of CRC cells by acting as ceRNAs, sponing miR-539, and increasing the expression of runt-related transcription factor 2 expression (33). Zhen et al. suggested that lncSNHG8 can function as a ceRNA, thereby promoting the multiplication, invasion, and migration of CRC cells by directly sponging with miR-663 (39). Xu et al. implied that lncSNHG6 function as an oncogene and could promote the cell cycle, enhance the invasion and migration ability of CRC cells by upregulating EZH1 expression via co binding and downregulation of miR-26a/b and miR-214 (40).

Some studies also revealed that lncSNHG could participate in the immune escape of tumor cells. For example, Zhou et al. revealed that lncSNHG4 could induce the immune escape of CRC cells *via* PD-1/PD-L1 activation and inducing CD4+ T cell apoptosis by sponging on miR-144-3p (13).

Several lncSNHG may induce chemotherapy resistance of CRC and promote cancer progression. For example, Ghasemi et al. validated that lncSNHG6 could drive the proliferation and drug resistance of CRC cells by upregulating the RAS and MAPK/AKT pathway (41). Saeinasab al. reported that lncSNHG15 may promote colon cancer and increases the resistance of CRC cells to 5-fluorouracil by interacting with apoptosis induced factor (42).

This study has some limitations. First, all the patients included were Asias; therefore, our conclusions can only represent the Asian population. Secondly, many original literatures only provided survival curve without HR value, which causes methodological bias to some extent. Finally, the number of patients in a single study is small, which can affect the overall results to some extent.

This is study is the first to explore the correlation between lncRNA SNHGs expression level and the prognosis of CRC. We revealed that high expression of lncRNA SNHGs is significantly related to poor CRC prognosis, and the expression level of lncRNA SNHGs may be used as a significant prognostic marker and potential therapeutic target of CRC.

## 5 Conclusion

High lncRNA SNHG expression implies worse prognosis for CRC, especially SNHG1. Additionally, lncRNA SNHG may function as potential therapeutic target for CRC. Therefore, an original study with higher quality is required to further support the consequence of this study.

## Data availability statement

All data generated or analyzed during this study are included in this published article or are available from the corresponding author on reasonable request.

## Author contributions

PL designed the study; PL and YL searched databases and performed literature screening; PL and JD extracted and analyzed the data; JM and WS evaluated the quality of included literature; PL, JD, YL and JM contributed to writing the manuscript. Final draft was approved by all the authors.
